# Enhanced recovery after bariatric surgery: a comprehensive survey-based analysis of ERABS actual clinical implementation in Italian bariatric centers

**DOI:** 10.1007/s13304-024-02009-9

**Published:** 2024-10-04

**Authors:** Lidia Castagneto-Gissey, Maria Francesca Russo, Annalisa Diddoro, Maurizio De Luca, Mario Musella, Giuseppe Navarra, Luigi Piazza, Marco Antonio Zappa, Marco Raffaelli, Nicola Di Lorenzo, Giovanni Casella, Collaborative Group, Altorio Settimo Fabrizio, Ambrosi Antonio, Andrea Lucchi, Andrea Porta, Baccari Paolo, Balani Alessandro, Barbato Domenico, Bardi Ugo, Battistoni Marco, Bellini Rosario, Berjaoui Abdul Halim, Bernante Paolo, Bertolani Lilia, Biagio Sodano, Bianchi Paolo Pietro, Boni Luigi, Bottino Vincenzo, Callari Cosimo, Caruso Francesco, Catarci Marco, Cavallaro Giuseppe, Cesari Maurizio, Ciampaglia Franco, Cobellis Luigi, Comaschi Marco, Corso Vittorio, Cristiano Stefano, Cuccurullo Diego, De Paoli Marco, Di Marzo Giancarlo, Di Paola Massimiliano, Docimo Ludovico, Donato Francesco Altomare, Enrico Facchiano, Fantola Giovanni, Finco Cristiano, Foletto Mirto, Gambetti Andrea, Gentileschi Paolo, Giuliano Sarro, Giuseppe Sarra, Giusto Pignata, Mario Guerrieri, La Malfa Giuseppe, Lattuada Ezio, Longoni Mauro, Lucchese Marcello, Manca Giuseppe, Marchesi Federico, Marinari Giuseppe Maria, Marzano Bernardo, Mastrandrea Giuseppe, Merola Giovanni, Moroni Roberto, Olmi Stefano, Paganini Alessandro M., Peri Andrea, Perrotta Nicola, Piccoli Micaela, Pierpaolo Cutolo, Pizza Francesco, Pizzi Mattia Edoardo Pietro, Potito Salatto, Rizzi Andrea, Rossetti Gianluca, Ruffo Giacomo, Scalambra Marco, Schettino Angelo Michele, Spampinato Marcello, Stipa Francesco, Vuolo Giuseppe

**Affiliations:** 1 Department of Surgery, Sapienza University of Rome, Viale Regina Elena, 324, 00161 Rome, Italy; 2 Dipartimento di Chirurgia Generale e Metabolica, Azienda ULSS5 Polesana, Ospedale di Rovigo, 45010 Rovigo, Italy; 3 Dipartimento di Scienze Biochimiche Avanzate, Università Degli Studi Di Napoli “Federico II”, 80138 Naples, Italia; 4 Policlinico Universitario “G. Martino” Messina, 98124 Messina, Italy; 5 UOC Chirurgia Generale e d’Urgenza, Arnas Garibaldi, 95123 Catania, Italy; 6 ASST Fatebenefratelli-Sacco, 20157 Milan, Italy; 7 Bariatric and Metabolic Surgery Unit, Fondazione Policlinico Universitario Agostino Gemelli IRCCS, Rome, Italy

**Keywords:** ERABS, ERAS, Bariatric–metabolic surgery, Sleeve gastrectomy, Roux-en-Y gastric bypass, One anastomosis gastric bypass

## Abstract

The escalating global prevalence of bariatric procedures necessitates an enhanced focus on optimizing perioperative care for improved patient outcomes. This study focuses on the implementation of Enhanced Recovery After Bariatric Surgery (ERABS) protocols in Italian bariatric centers to optimize perioperative care. An online survey comprising 19 items was conducted in October 2023, targeting managing surgeons in 139 registered bariatric centers. This survey explored geographic and center-type variations, knowledge, and application of ERABS protocols, along with perioperative, intraoperative, and postoperative practices. Statistical analysis employed included one-way ANOVA and Tukey post hoc tests. Responses from 72 centers (51.8%) revealed a strong awareness of ERABS protocols among managing surgeons. Adherence rates varied geographically (46.3–63%) and among center types (60.8–56.3%). Perioperative practices, including the abandonment of antibiotics for perioperative prophylaxis by 77.5% of surgeons and the adoption of the TAP block technique in 69% of centers, aligned well with ERABS recommendations. However, inconsistencies were noted in abdominal drain usage (61.5% after SG; 70.5% after RYGB/OAGB), with a substantial percentage not adhering to ERABS guidelines. Intraoperative habits generally adhered to ERABS protocols, yet preferences for certain tests, particularly the methylene blue test (70.4%), deviated. Postoperative practices displayed positive trends, with early reintroduction of oral feeding (71.8%) and opioid-free pain management (71.8%). Variations existed in discharge timing and patient monitoring, indicating areas for further improvement. This study offers a comprehensive snapshot of ERABS protocol adherence in Italy, emphasizing the positive trend toward optimizing recovery and reducing patient stress. Despite variations, a majority of centers demonstrated commitment to ERABS principles. Ongoing education, interdisciplinary collaboration, and nationwide dialogue are essential for standardizing ERABS protocols and advancing bariatric–metabolic surgery outcomes in Italy.

**Supplementary Information:**

The online version contains supplementary material available at 10.1007/s13304-024-02009-9.

## Introduction

The worldwide prevalence of bariatric procedures has seen a consistent uptrend over the past 2 decades [1]. This surge can be attributed to advancements in surgical proficiency, the standardization of preoperative protocols, and meticulous candidate selection, leading to notably enhanced outcomes and minimal reported complications [2]. The paradigm shift toward minimally invasive techniques has revolutionized bariatric–metabolic surgery. The subsequent logical stride is to devise strategies for optimizing perioperative care for bariatric patients, as the demand for surgical interventions continues to grow.

Enhanced Recovery After Surgery (ERAS) represents a strategic protocol designed to mitigate perioperative stress for patients, consequently culminating in enhanced postoperative outcomes concerning morbidity and length of hospital stay. The controversy surrounding ERAS protocols and their implementation constitutes a subject of considerable interest and contention within surgical practice. The initial evidence-based consensus protocol for individuals undergoing colonic resection was promulgated by the ERAS Study Group in 2005, thereby laying the foundation for subsequent developments in this domain [3].

Subsequent to this seminal milestone, the ERAS Society has diligently facilitated the application of these guidelines, implementing bespoke protocols for an array of surgical disciplines, notably including bariatric–metabolic surgery under the acronym ERABS. In 2016, the initial iteration of the Guidelines for Perioperative Care in Bariatric Surgery was published [4], and subsequently, an updated version was issued in 2021 [5].

The burgeoning prevalence of bariatric–metabolic surgery in Italy, coupled with the escalating complexity of bariatric patients, has underscored the imperative for a standardized and optimized approach to management. Recognizing this need, in 2022, The Italian Society of Obesity Surgery and Metabolic Diseases (SICOb) and the Italian Society of Anesthesia, Analgesia, Resuscitation, and Intensive Care (SIAARTI) collaboratively formulated the first Italian consensus statement for enhanced recovery after bariatric–metabolic surgery. Within this paper, a compendium of 25 recommendations was proffered, encompassing facets of preoperative evaluation and care, intraoperative management, postoperative directives, and discharge protocols [6].

The SICOb guidelines underwent a comprehensive update in 2023, presenting robust endorsements for a peri- and intra-operative management model that integrates some or all components delineated in ERABS protocols [7]. The adoption of the ERABS approach, as per these guidelines, portends improved recovery outcomes, diminished length of hospital stay, and reduced patient stress, all without concomitant increase in complications [5, 6]. The articulation of a national statement assumes paramount significance as it constitutes the key to achieving standardization across Italian bariatric surgical centers.

Despite the well-established feasibility and safety profile of the ERABS approach and the ready availability of national guidelines, the effective application of these protocols in Italian clinical practice, particularly in the context of bariatric–metabolic surgery, remains conspicuously absent from existing literature, contrasting with reports from other surgical domains [8].

The primary aim of this study is to investigate the actual implementation of ERAS protocols within Italian bariatric centers, thereby providing an objective assessment of the current state of adherence to these principles.

## Methods

### Study design

This was an online survey that consisted of 19 items. The online questionnaire link was shared via e-mail using the SICOb Network database on October 28, 2023 and was open for a period of 30 days. To avoid duplicate data, only one referral person from each Italian bariatric center was selected. The survey was anonymously administered to 139 centers registered with SICOb as an online survey in October 2023 and was directed specifically to the center's managing surgeon.

**Fig. 1:**
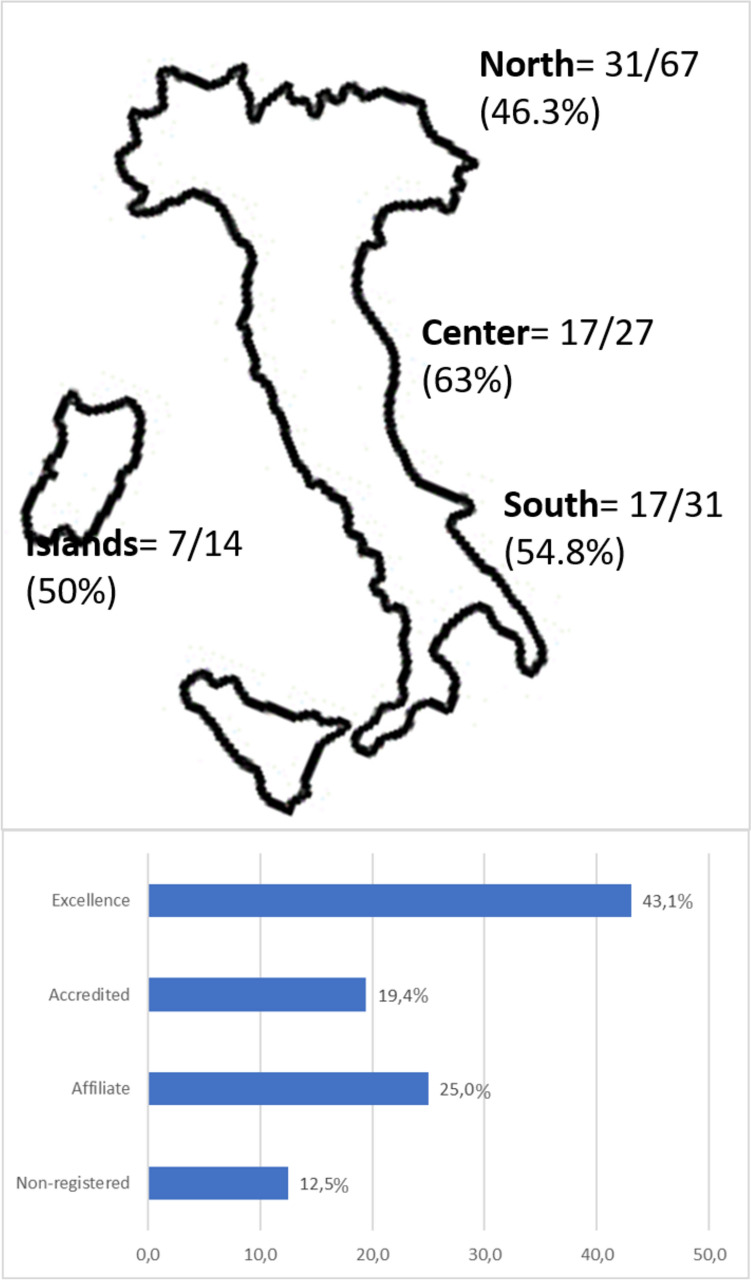
Geographic distribution of Italian bariatric centers and proportion between the number of responding and non-responding centers that participated in the survey. Proportion of participating centers divided by volume of cases per year (i.e., > 100 procedures/year, excellence center; > 50 procedures/year, accredited center; > 25 procedures/year, affiliated center)

According to the latest SICOb investigation, the Society has reported 139 registered bariatric–metabolic surgery centers, with a total of 23,501 surgical and endoscopic procedures performed in 2022. These centers are categorized by geographical area (North, Center, South, and Islands) and number of surgical procedures performed per year (excellence center: > 100 procedures/year; accredited center: > 50 procedures/year; affiliated center: > 25 procedures/year; non-registered center: centers not meeting all SICOb criteria for endorsement regardless of number of surgical procedures performed). Characteristics for each center type and geographic distribution of centers are illustrated in Fig. 1.

Guided by the National consensus statement for ERABS, we developed a questionnaire consisting of 19 items (Table 1). This questionnaire explores the peri- and intra-operative management of bariatric patients undergoing Sleeve Gastrectomy (SG), Roux-en-Y Gastric Bypass (RYGB), and One Anastomosis Gastric Bypass (OAGB), which collectively constitute the three most frequently performed procedures in Italy (57%, 12%, and 13%, respectively) according to the National Registry.

**Table 1:** Survey questions and answer options

Question	Answer choice
1. Are you familiar with the Enhanced Recovery After Surgery (ERAS) protocols applied to bariatric surgery (ERABS)?	Yes No Not precisely
2. Regarding the ERABS protocol:	I try to apply them systematically I take them into account but evaluate them on a case-by-case basis I do not apply them I apply the protocols imposed by my Hospital
3. Do you use a specific checklist for its application?	Yes No
4. Do you resort to antibiotic therapy beyond perioperative prophylaxis in patients undergoing standard bariatric surgery?	Yes No
5. Do you use the TAP block technique for pain control?	Yes No
6. Do you routinely use the following in patients undergoing sleeve gastrectomy?	Nasogastric Tube Yes/No Abdominal Drain Yes/No Urinary Catheter Yes/No
7. Do you routinely use the following in patients undergoing gastric bypass (RYGB/OAGB)?	Nasogastric Tube Yes/No Abdominal Drain Yes/No Urinary Catheter Yes/No
8. Do you routinely perform an intraoperative methylene blue test to check the integrity of the staple line/anastomosis?	Yes No I perform another type of test
9. Do patients undergoing bariatric surgery routinely resume oral intake?	Within 24 h after surgery Beyond 24 h after surgery Beyond 72 h after surgery
10. In patients undergoing bariatric surgery, do you routinely use opioid analgesics in single bolus injection or continuous infusion (e.g., elastomeric pump) for postoperative pain control?	Yes No
11. Generally, when does mobilization occur after bariatric surgery?	Within 6 h post-surgery After 6 h but within 24 h post-surgery Beyond 24 h post-surgery
12. Do you routinely perform a pre-discharge radiological check with a contrast study (e.g., upper GI swallow test) in patients undergoing bariatric surgery?	Yes No I perform another type of test (e.g., postoperative methylene blue)
13. On which postoperative day do you discharge patients undergoing sleeve gastrectomy with a regular postoperative course?	Day 1 Day 2 Days 3–4 Day 5 or later
14. On which postoperative day do you discharge patients undergoing gastric bypass (RYGB/OAGB) with a regular postoperative course?	Day 1 Day 2 Days 3–4 Day 5 or later
15. Do you engage in post-discharge telephone monitoring of patients undergoing bariatric surgery?	Yes No

Results are presented as percentages, at first encompassing the overall number of centers and subsequently separately by both geographic area and center type.

### Statistical analysis

A one-way ANOVA was performed to evaluate the relationship between groups by type and location and the items submitted. Tukey’s post hoc test was used to perform multiple comparisons between groups. *P* < 0.005 was considered statistically significant. All statistical analyses were carried out with SPSS Statistics v.27.0. (IBM Corp. Released 2020. IBM SPSS Statistics for Windows, Version 27.0. Armonk, NY: IBM Corp).

## Results

### Response rates and geographic distribution

In October 2023, 72 out of a total of 139 bariatric centers (51.8%) responded to the online survey proposed, representative of more than 15,000 surgical procedures performed in Italy each year. Different adhesion rates were observed based on geographic distribution: 46.3% in the North, 63% in the Center, 54.8% in the South, and 50% in the Islands. Figure 1 illustrates the proportion between the number of responding and non-responding centers.

### Center-type variations

**Fig. 2:**
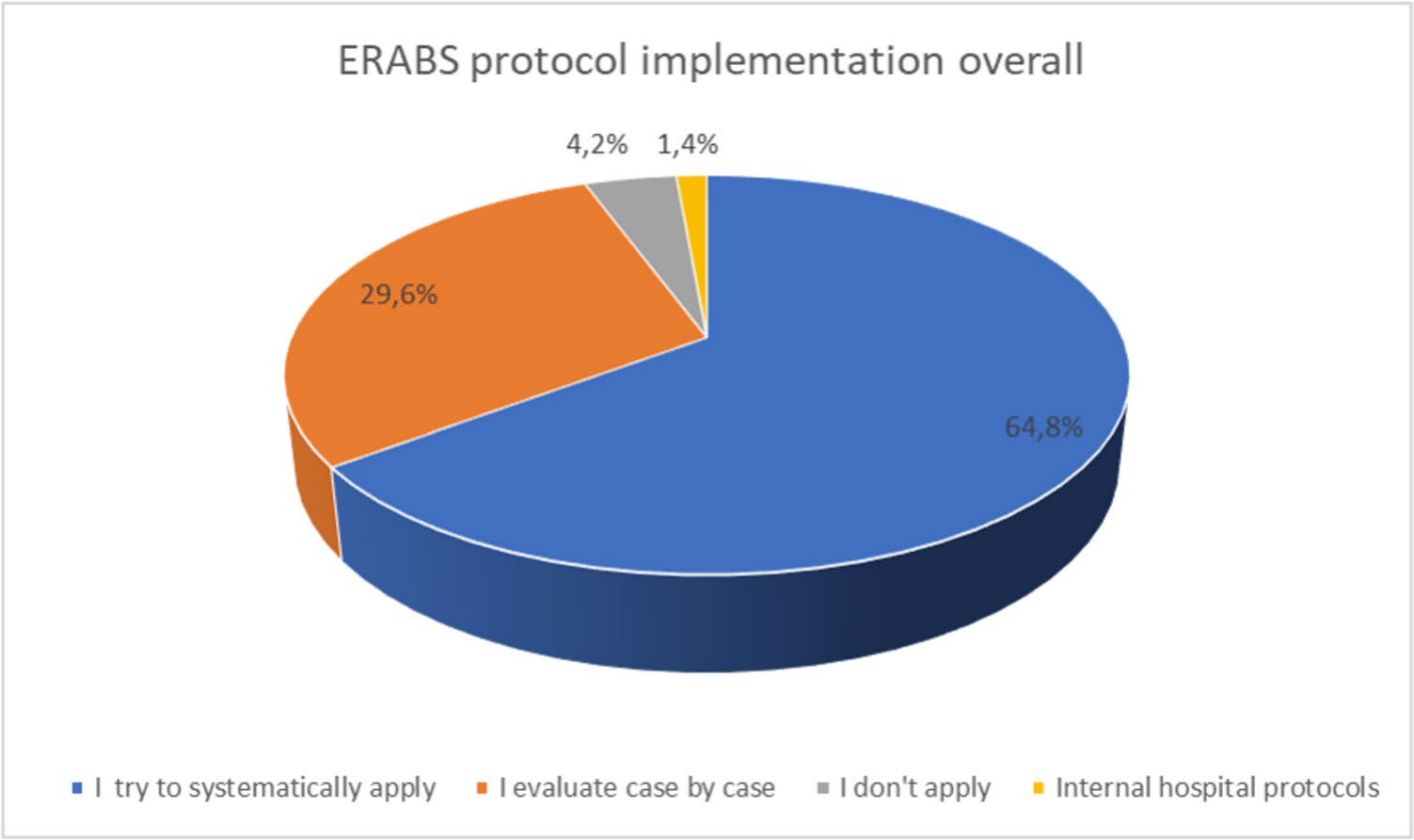
Overall ERABS protocol implementation across bariatric centers participating to the survey

The adhesion rate per center type was 60.8% for excellence centers, 51.9% for accredited centers, and 56.3% for affiliated centers. Nine (31%) non-registered centers (registered with SICOb but not meeting the criteria for endorsement) responded to the survey as well (Fig. 2).

### Knowledge and application of ERABS protocols

Concerning the totality of centers, the majority of the centers’ managing surgeons are well informed about the ERABS protocol, with only 2 (2.8%) having partial knowledge. The majority of centers attempt to apply (64.8%) or evaluate the application of the protocol on a case-by-case basis (29.6%). However, less than half (46.5%) of centers use a specific checklist for ERABS protocol application (question 3), contrasting with the previous answers. In excellence centers, this percentage rises to 58.1%, while it remains under 50% in all the other categories (Tables 2–3).

**Table 2:** Characteristics of bariatric centers responding to the survey

ERAS protocol implemented		
	*n* = 71	%
Yes	70	97.2
No	2	2.8
Hospital type		
University Hospital	3	4.2
Public Hospital	43	60.6
Private Hospital	15	21.1
Other	10	14.1
Type of center		
Excellence	31	43.7
Accredited	13	18.3
Affiliated	18	25.3
Non-registered	9	12.7
Geographic Localization		
North	30	42.3
South	17	23.9
Center	17	23.9
Islands	7	9.9

**Table 3:** Overall compliance denotes the full implementation of ERABS items, categorized by center type and geographical location

Compliance	%
Overall (*n* = 71)	58.4
By type of center	
Excellence (*n* = 31)	62.1
Affiliated (*n* = 18)	47.7
Accredited (*n* = 13)	66.9
Non-registered (*n* = 9)	60.1
By geographic localization	
North (*n* = 30)	65.1
South (*n* = 17)	51.9
Center (*n* = 17)	51.5
Islands (*n* = 7)	69.7

No significant differences were found in terms of compliance between center type and geographic localization

### Perioperative practices

The use of antibiotics over perioperative prophylaxis (question 4) has been abandoned by 77.5% of the surgeons, and the TAP block technique (question 5) is adopted in 69% of centers as a perioperative pain control strategy (Tables 4, 5).

**Table 4:** Compliance with ERABS items categorized by type of bariatric center, with overall compliance displayed as a reference point across all centers

	Excellence (*n* = 31)	Accredited (*n* = 13)	Affiliated (*n* = 18)	Non-registered (*n* = 9)	Overall
Dedicated checklist, *n* (%)	18 (58)	5 (38.4)	8 (44.4)	2 (22.2)	33 (46.5)
Postoperative antibiotic therapy, *n* (%)	23 (74.2)	11 (84.6)	14 (77.7)	7 (77.7)	55 (77.5)
Neuromuscular blockade	21 (67.7)	8 (61.5)	13 (72.2)	7 (77.7)	49 (69)
Analgesia opioid sparing, *n* (%)	23 (74.2)	10 (76.9)	14 (77.7)	9 (100)	51 (71.8)
Nasogastric Tube in LSG, *n* (%)	28 (90.3)	13 (100)	17 (94.4)	9 (100)	67 (94.4)
Abdominal Drainage in LSG, *n* (%)	15 (48.4)	7 (53.8)	4 (22.2)	1 (11.1)	27 (38)
Bladder catheter in LSG, *n* (%)	28 (90.3)	13 (100)	12 (66.6)	9 (100)	62 (87.3)
Nasogastric Tube in RYGB/OAGB, *n* (%)	25 (80.6)	11 (84.6)	15 (83.3)	9 (100)	61 (85.9)
Abdominal Drainage in RYGB/OAGB, *n* (%)	12 (38.7)	6 (46.1)	3 (16.6)	0 (0)	21 (29.5)
Bladder catheter in RYGB/OAGB, *n* (%)	25 (80.6)	13 (100)	7 (38.8)	8 (88.8)	53 (74.6)
Methylene blue test, *n* (%)	9 (29)	3 (23.1)	2 (11.1)	5 (55.5)	19 (26.7)
Upper gastrointestinal series prior discharge, *n* (%)	13 (41.9)	10 (76.9)	2 (11.1)	1 (11.1)	20 (28.1)
Early mobilization (within 6 h), *n* (%)	15 (48.4)	11 (84.6)	10 (55.5)	6 (66.6)	42 (59.1)
Early oral liquid diet resumption (within 24 h), *n* (%)	23 (74.2)	9 (69.2)	11 (61.1)	8 (88.8)	51 (71.8)
Early discharge in LSG (POD 1–2), *n* (%)	16 (51.6)	7 (53.8)	3 (16.6)	4 (44.4)	30 (42.2)
Early discharge in RYGB/OAGB (POD 1–2), *n* (%)	15 (48.4)	6 (46.1)	2 (11.1)	3 (33.3)	26 (36.6)
Remote monitoring after surgery, *n* (%)	20	5	9	4	38 (53.5)

Data are presented as numbers and percentages

**Table 5:** Compliance with ERABS items categorized by geographic location, with overall compliance displayed as a reference point across all centers

	North (*n* = 30)	South (*n* = 17)	Center (*n* = 17)	Islands (*n* = 7)	Overall
Dedicated checklist, *n* (%)	15 (50)	8 (47)	6 (35.3)	4 (57.1)	33 (46.5)
Postoperative antibiotic therapy, *n* (%)	25 (83.3)	10 (58.8)	14 (82.3)	6 (85.7)	55 (77.5)
Neuromuscular blockade, *n* (%)	21 (79)	9 (52.9)	14 (82.3)	5 (71.4)	49 (69)
Analgesia opioid sparing, *n* (%)	28 (93.3)	9 (52.9)	11 (64.7)	3 (42.8)	51 (71.8)
Nasogastric Tube in LSG, *n* (%)	29 (96.6)	15 (88.2)	16 (94.1)	7 (100)	67 (94.4)
Abdominal Drainage in LSG, *n* (%)	12 (40)	4 (23.5)	5 (29.4)	6 (85.7)	27 (38)
Bladder catheter in LSG, *n* (%)	29 (96.6)	13 (76.4)	13 (76.4)	7 (100)	62 (87.3)
Nasogastric Tube in RYGB/OAGB, *n* (%)	27 (90)	13 (76.4)	14 (82.3)	7 (100)	61 (85.9)
Abdominal Drainage in RYGB/OAGB, *n* (%)	10 (33.3)	14 (82.3)	4 (23.5)	4 (57.1)	21 (29.5)
Bladder catheter in RYGB/OAGB, *n* (%)	25 (83.3)	11 (64.7)	11 (64.7)	6 (85.7)	53 (74.6)
Methylene blue test, *n* (%)	11 (36.6)	4 (23.5)	2 (11.7)	2 (28.5)	19 (26.7)
Upper gastrointestinal series prior discharge, *n* (%)	10 (33.3)	4 (23.5)	4 (23.5)	2 (28.5)	20 (28.1)
Early mobilization (within 6 h), *n* (%)	20 (66.6)	11 (64.7)	5 (29.4)	4 (57.1)	42 (59.1)
Early oral liquid diet resumption (within 24 h), *n* (%)	23 (76.6)	8 (47)	13 (76.4)	7 (100)	51 (71.8)
Early discharge in LSG (POD 1–2), n (%)	17 (56.6)	4 (23.5)	5 (29.4)	4 (57.1)	30 (42.2)
Early discharge in RYGB/OAGB (POD 1–2), *n* (%)	15 (50)	4 (23.5)	4 (23.5)	3 (42.8)	26 (36.6)
Remote monitoring after surgery, *n* (%)	15 (50)	9 (52.9)	8 (47)	6 (85.7)	38 (53.5)

Data are presented as numbers and percentages

### Intraoperative habits and testing

**Fig. 3:**
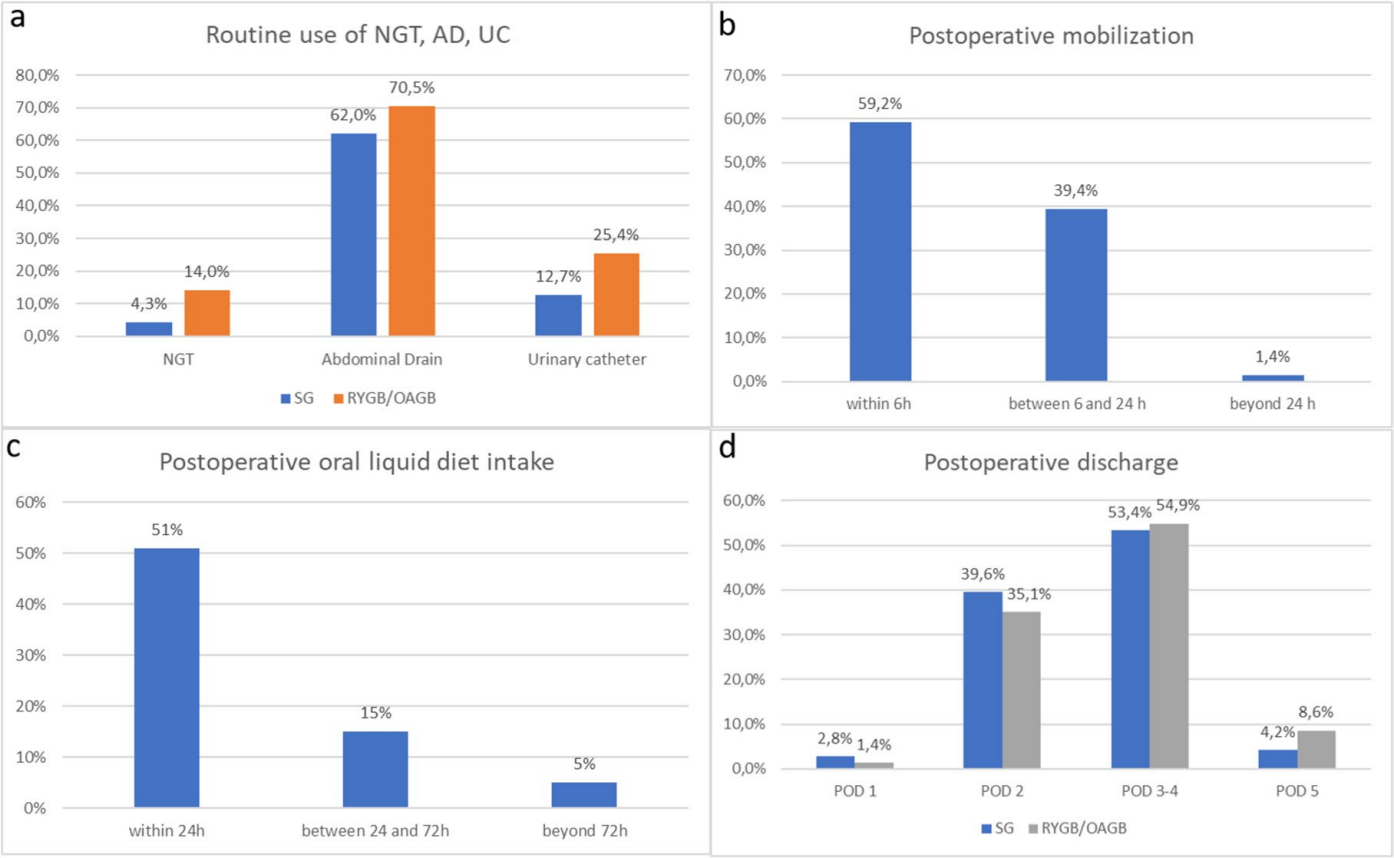
Comparison of **a** nasogastric tube, abdominal drain, and urinary catheter use after SG and RYGB/OAGB; **b** postoperative mobilization across all procedures; **c** postoperative oral intake across all procedures in the surveyed centers; **d** postoperative discharge days following RYGB/OAGB and SG

Intraoperative habits of surgeons regarding the positioning of a nasogastric tube (NGT), abdominal drain (DRG), and urinary catheter (UC) during SG and RYGB/OAGB were investigated separately (questions 6 to 11). The use of intraoperative methylene blue test in both procedures was also explored (question 12). Figure 3 illustrates the application of ERABS protocols concerning the use of the UC and NGT which seem to be widely applied (no routine positioning of NGT in 96% and 86% and no routine positioning of UC in 87.3% and 74.6% in SG and RYGB, respectively). However, there is a poor application of the ERABS protocol in terms of abdominal drain usage. In fact, the routine positioning of abdominal drains is not expected as per protocol; however, abdominal drains were not used in 38% of cases in the SG group and 29.5% in the RYGB group (Fig. 3, Tables 4, 5).

The intraoperative employment of a methylene blue test, compared with other intraoperative tests or no intraoperative test, was assessed. The majority of surgeons prefer to perform an intraoperative test in the majority of cases (methylene blue 70.4% or other kinds of tests 1.4%) despite ERABS recommendations.

### Postoperative management

The early reintroduction of oral feeding with a liquid diet, as recommended by both ERAS and ERABS protocols, was investigated in question 13. Three time intervals where oral feeding was reintroduced were identified: within 24 h (71.8%), 24–72 h (21.1%), and after 72 h (7.1%) (Fig. 3c).

Postoperative pain is managed in an opioid-free approach in 71.8% of centers, with a higher rate in the North of Italy (93.3%) and a lower rate in the Islands (42.9%). Postoperative mobilization mainly occurs within 24 h after surgery (98.6%), with 59% within 6 h from surgery (Tables 4, 5).

**Fig. 4:**
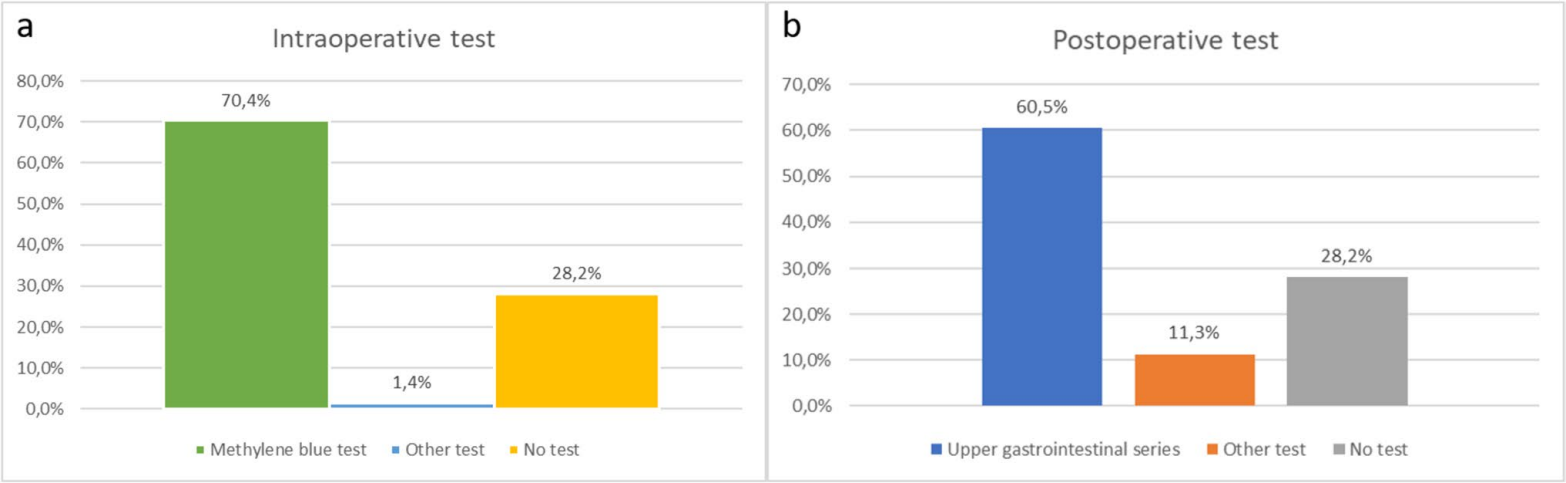
Comparison of **a** intraoperative leak test; **b** postoperative pre-discharge test

In question 16, the employment of pre-discharge radiologic tests is investigated. Despite guidelines recommending no radiologic check before discharge, the clinical practice differs: 60.5% of centers perform a pre-discharge radiologic test, and 11.3% resort to other tests (Fig. 4).

Timing of discharge was analyzed separately for SG and OAGB/RYGB by questions 17 and 18; results are illustrated in Fig. 3d. In the majority of centers, patients are discharged on postoperative day (POD) 3 or 4 (53.4% SG and 54.9% RYGB/OAGB), followed by POD 2 (39.6% SG and 35.1% RYGB/OAGB). The ERABS protocol considers the discharge on POD 1 or 2 as feasible and safe if there are no uncertain clinical or serological features, assuming that correct information and education of patients regarding signs and symptoms of possible complications and providing them with a rapid way to contact medical staff is completed.

ERABS protocols also include procedures and indications after discharge for the best and safest postoperative course for patients. This is not always possible, as emerges from question 19. Only 53.4% of centers offer a phone call monitoring service dedicated to patients, with dedicated bariatric physicians or nurses who can complete the health check.

### Comparison between center types

There was a statistically significant difference between groups, namely, excellence, affiliated, accredited, and non-registered centers, regarding the item “Do you usually administer postoperative opiates (bolus or continuous IV infusion) to patients undergoing bariatric–metabolic surgery?” as determined by one-way ANOVA (*F*(3,67) = 2.844, *p* = 0.044). A Tukey post hoc test revealed that affiliated centers were superior to non-registered centers (*p* = 0.032) (Supplementary Tables 1 and 2).

Furthermore, regarding the item “Which postoperative day are patients discharged after SG?” a statistically significant difference was found (*F*(3,67) = 3.280, *p* = 0.026). Tukey post hoc analysis showed that affiliated centers discharged patients earlier after SG when compared to accredited centers (*p* = 0.023) (Supplementary Table 2).

Also, the item “Which postoperative day are patients discharged after RYGB/OAGB” showed a statistically significant difference when running a one-way ANOVA (*F*(3,67) = 3.712, *p* = 0.016). Again, a Tukey post hoc test revealed that excellence centers were the ones discharging patients earlier after RYGB/OAGB than the others (*p* = 0.014) (Supplementary Table 3).

### Comparison between regions

There was a statistically significant difference between groups, namely, Northern regions, Southern regions, Central regions, and Islands, regarding the item “When do patients undergoing bariatric–metabolic surgery routinely resume an oral liquid diet?” as determined by one-way ANOVA (F(3,67) = 3.008, p = 0.036). From a Tukey post hoc test appeared that in centers located in Southern regions water fast was resumed before than in centers located on the Islands (p = 0.041) revealing that affiliated centers were superior to non-registered centers (p = 0.032) (Supplementary Tables 4–5).

Furthermore, regarding the item “Do you usually administer postoperative opiates (bolus or continuous IV infusion) to patients undergoing bariatric–metabolic surgery?” a statistically significant difference was found between groups (*F*(3,67) = 5.093, *p* = 0.003). Tukey post hoc analysis showed that centers located in Northern Italy were more likely to administer opiates postoperatively compared to centers located in the South and on the Islands (*p* = 0.012 and *p* = 0.027, respectively) (Supplementary Table 6).

## Discussion

This survey allows us to analyze the habits of bariatric surgeons nationwide, using a sample that effectively represents all geographic areas and various types of centers. We have assessed only the main aspects of the application of ERABS protocols and have observed that some recommendations have a higher penetration in clinical practice than others.

The results of our study provide a comprehensive snapshot of the current state of adherence to ERABS protocols in Italian bariatric centers. The survey, conducted in October 2023, garnered responses from 72 out of 139 centers, representing a response rate of 51.8% and capturing a significant portion of the surgical landscape with over 15,000 procedures performed annually.

One notable finding is the variability in adherence rates based on geographic distribution and center type. Adherence rates varied from 46.3% in the North to 63% in the Center, 54.8% in the South, and 50% in the Islands. Similarly, different adherence rates were observed among center types, with excellence centers showing a higher rate (60.8%) compared to accredited (51.9%) and affiliated centers (56.3%).

The majority of managing surgeons across all centers demonstrated a strong awareness of the ERABS protocol, with only a minimal 2.8% reporting partial knowledge. However, the application of the protocol varied, with 64.8% attempting to apply and 29.6% evaluating its application on a case-by-case basis. Notably, excellence centers exhibited a higher rate of using a specific checklist for ERABS protocol application (58.1%) compared to other center types.

Several perioperative practices aligned with ERABS recommendations, including the abandonment of antibiotics for perioperative prophylaxis by 77.5% of surgeons and the adoption of the TAP block technique in 69% of centers for pain control. While the use of nasogastric tubes and urinary catheters aligned with ERABS protocols, a notable gap was identified in the routine use of abdominal drains, deviating from recommended practices. The majority of surgeons favored intraoperative tests, particularly the methylene blue test, despite ERABS recommendations, showcasing a preference for established practices.

The early reintroduction of oral feeding and opioid-free postoperative pain management were in accordance with ERABS guidelines, reflecting positive adherence trends. However, variations were observed in the timing of discharge, radiologic testing, and postoperative patient monitoring, indicating areas for further improvement and standardization.

Statistically significant differences were noted in postoperative opiate administration and discharge practices among excellence, accredited, affiliated, and non-registered centers. Similarly, variations were observed in liquid diet resumption and postoperative opiate administration across different regions, emphasizing the influence of center type and geographic location on bariatric practices.

It is important to emphasize that ERAS (and ERABS) is an approach that begins at the patient's home and concludes there. Elements such as patient education, territorial assistance facilities, and the availability of care services are crucial in allowing and supporting surgeons in applying ERABS recommendations [9–11]. On the other hand, it is essential to consider that the application of the ERAS approach in bariatric–metabolic surgery has recently been introduced and codified in Italy, encountering the established practices of surgeons and other physicians involved in managing bariatric patients.

With a primary focus on ensuring clinical safety, ERAS protocols play a crucial role in expediting the recovery of patients undergoing bariatric–metabolic surgery by especially minimizing postoperative nausea and vomiting and alleviating postoperative pain, thereby reducing hospital stays. Noteworthy is the considerable reduction in medical costs and resource consumption, aside from the shortened hospitalization periods [9, 10]. The implementation of effective preoperative education and counseling contributes to easing perioperative anxiety and enhancing the compliance of bariatric–metabolic surgery patients [11]. Moreover, beyond prioritizing and maintaining the physical and psychological needs of patients, ERAS optimizes the quality of medical services, leading to enhanced patient satisfaction [12]. From the perspective of healthcare providers and institutions, a collaborative, multidisciplinary approach can dismantle disciplinary barriers, significantly boosting efficiency. ERAS implementation not only lightens the load on healthcare providers but also diminishes the demand for hospital beds, ultimately increasing turnover rates [5, 13, 14]. This holds particular significance for regions facing scarcity in medical resources.

In contemporary bariatric–metabolic surgery, the adoption of numerous elements from the ERAS protocol has gained widespread acceptance and demonstrated connections to reduced perioperative complications and accelerated recovery. Nevertheless, the supporting evidence for several ERAS interventions within the context of bariatric procedures is somewhat limited, necessitating the consideration of extrapolating evidence-based practices from other surgical domains.

The adoption of this approach will likely be gradual, but even a partial implementation of the indications included in the protocol is useful in achieving the optimization of recovery and reducing stress for patients [5]. Adherence to ERABS is a developing process in Italy, involving not only surgeons but also anesthesiologists, nutritionists, psychologists, nurses, and all healthcare workers. Each of these healthcare professionals should acquire comprehensive knowledge about the protocols and be skilled in their safe and correct application.

## Conclusions

In conclusion, our study sheds light on the complex landscape of ERABS protocol adherence in Italian bariatric centers. While certain practices align well with established guidelines, variations persist, indicating a need for ongoing education, standardization efforts, and interdisciplinary collaboration. The results provide valuable insights for bariatric surgeons, healthcare professionals, and policymakers to address challenges and enhance the implementation of ERABS protocols for improved patient outcomes. Continued research and dialogue within the bariatric community will be crucial for advancing the field and achieving a more standardized and optimized approach to bariatric–metabolic surgery in Italy.

## Supplementary Information

Below is the link to the electronic supplementary material.

Supplementary file1 (DOCX 16 KB)

## Data Availability

Raw data will be shared upon reasonable request.
