# Supplementary material for: Enhanced recovery after bariatric surgery: a comprehensive survey-based analysis of ERABS actual clinical implementation in Italian bariatric centers
Source: Updates Surg. 2024 Oct 4;77(7):1967–77. doi: 10.1007/s13304-024-02009-9 (PMC12540566; doi:10.1007/s13304-024-02009-9)
Supplement: Supplementary file 1 — Supplementary file1 (DOCX 16 KB) [file 13304_2024_2009_MOESM1_ESM.docx]

**Supplementary table 1**. Do you usually administer postoperative opiates (bolus or continuous IV infusion) to patients undergoing bariatric surgery?

|  | Sum of squares | df | Mean Square | F | Sig. |
| --- | --- | --- | --- | --- | --- |
| Between Groups | *1.623* | *3* | *.541* | *2.844* | *.044* |
| Within groups | *12.743* | *67* | *.190* |  |  |
| Total | *14.366* | *70* |  |  |  |

**Supplementary table 2**. Which postoperative day are patients undergoing sleeve gastrectomy discharged?

|  | Sum of squares | df | Mean Square | F | Sig. |
| --- | --- | --- | --- | --- | --- |
| Between Groups | *3.477* | *3* | *1.159* | *3.280* | *.026* |
| Within groups | *23.678* | *67* | *.353* |  |  |
| Total | *27.155* | *70* |  |  |  |

**Supplementary table 3**. Which postoperative day are patients undergoing RYGB/OAGB discharged?

|  | Sum of squares | df | Mean Square | F | Sig. |
| --- | --- | --- | --- | --- | --- |
| Between Groups | *4.103* | *3* | *1.368* | *3.712* | *.016* |
| Within groups | *24.686* | *67* | *.368* |  |  |
| Total | *28.789* | *70* |  |  |  |

**Supplementary table 4**.

Multiple comparison analysis by type of centre

| Tukey HSD |  |  | Mean Difference | Std. Error | Sig. | 95% Confidence Interval  Lower Bound Upper Bound |
| --- | --- | --- | --- | --- | --- | --- |
| Do you usually administer post operative opiates (bolus or continuous IV infusion) to patients undergoing bariatric surgery? | *Affiliated* | *Non-registered* | *.50000** | *.17804* | *.0032* | *.0309 .9691* |
| Which postoperative day are patients undergoing sleeve gastrectomy discharged? | *Affiliated* | *Accredited* | *.63675** | *.21637* | *0.023* | *.0667 12.068* |
| Which postoperative day are patients undergoing RYGB/OAGB discharged? | *Excellence* | *Affiliated* | *-.56272** | *.17987* | *0.014* | *-10.366 -.0888* |

**Supplementary table 5**. When do patients undergoing bariatric surgery routinely resume water fast?

|  | Sum of squares | df | Mean Square | F | Sig. |
| --- | --- | --- | --- | --- | --- |
| Between Groups | *1.705* | *3* | *.568* | *3.008* | *.036* |
| Within groups | *12.661* | *67* | *.189* |  |  |
| Total | *14.366* | *70* |  |  |  |

**Supplementary table 6**. Multiple comparison analysis by geographic localization

| Tukey HSD |  |  | Mean Difference | Std. Error | Sig. | 95% Confidence Interval  Lower Bound Upper Bound |
| --- | --- | --- | --- | --- | --- | --- |
| When do patients undergoing bariatric surgery routinely resume water fast? | *Southern* | *Islands* | *-.52941** | *.19522* | *.041* | *-10.438 -.0151* |
| Do you usually administer post operative opiates (bolus or continuous IV infusion) to patients undergoing bariatric surgery? | *Northern* | *Southern*  *Islands* | *-.40392**  *-.50476** | *.12685*  *.17450* | *.012*  *.027* | *-.7381 -.0697*  *-.9669 -.0426* |
